# Chronic traumatic encephalopathy: Diagnostic updates and advances

**DOI:** 10.3934/Neuroscience.2022030

**Published:** 2022-12-19

**Authors:** Kevin Pierre, Vanessa Molina, Shil Shukla, Anthony Avila, Nicholas Fong, Jessica Nguyen, Brandon Lucke-Wold

**Affiliations:** 1 University of Florida Department of Radiology, Gainesville 32603, Florida, USA; 2 Sam Houston State University of Osteopathic Medicine, Conroe 77304, Texas, USA; 3 University of Florida Department of Neurosurgery, Gainesville 32603, Florida, USA

**Keywords:** chronic traumatic encephalopathy, diagnostics, work up, management

## Abstract

Chronic traumatic encephalopathy (CTE) is a progressive neurodegenerative disease that occurs secondary to repetitive mild traumatic brain injury. Current clinical diagnosis relies on symptomatology and structural imaging findings which often vary widely among those with the disease. The gold standard of diagnosis is post-mortem pathological examination. In this review article, we provide a brief introduction to CTE, current diagnostic workup and the promising research on imaging and fluid biomarker diagnostic techniques. For imaging, we discuss quantitative structural analyses, DTI, fMRI, MRS, SWI and PET CT. For fluid biomarkers, we discuss p-tau, TREM2, CCL11, NfL and GFAP.

## 1. Introduction

Chronic traumatic encephalopathy (CTE) is a neurodegenerative disease secondary to repetitive mild traumatic brain injury (mTBI), including concussions and sub-concussive impacts, resulting in long-term issues with cognition, behavior and mood [1]–[7]. CTE was initially recognized in boxers who developed symptoms like ataxia, memory loss and personality change, and it was coined as the “punch drunk” syndrome or “dementia pugilistica” [1],[3],[4],[8]–[10]. Over time, it became evident that CTE also affected military personnel, domestic violence victims and those participating in contact sports like football, ice hockey, professional wrestling, rugby, soccer and boxing [1]–[4],[8].

Neurodegeneration and symptoms in CTE progress even in the absence of further traumatic insults [2],[6],[11],[12]. mTBI is thought to trigger an inflammatory cascade and lead to blood brain barrier permeability, axonal injury and micro-hemorrhages [13]–[16]. As a result, there is deposition of pathogenic proteins, including the pathogenic cis-isoform of p-tau, which, through the process termed cistauosis, catalyzes conversion of normal into pathogenic tau [2],[17]–[21]. As such, CTE develops in pathological stages with worsening depositions of p-tau, neurofibrillary tangles and brain atrophy in similar but distinct fashions as other neurodegenerative diseases like Alzheimer's disease [2].

The current gold standard diagnosis for CTE is post-mortem pathological examination. Trauma encephalopathy syndrome was proposed to help diagnose patients with CTE. This criterion consists of a history of repetitive brain injury, persistent symptoms over a year and an absence of comorbidities that may also account for the symptoms. Also present should be a cognitive, behavioral or mood impairment in the presence of progressive decline over more than a year, impulsivity or headaches [2],[22]–[24]. However, patients with CTE present differently and there is no consensus on a single, best set of clinical or research diagnostic criteria [2],[5],[6],[25]. Therefore, there is increasing investigation of adjunctive non-invasive diagnostic modalities. In this paper, we review the recent advances in the use of neuroimaging and fluid biomarkers for early CTE detection.

### 1.1. Diagnostic Imaging

#### 1.1.1. Magnetic Resonance Imaging

Magnetic resonance imaging (MRI) produces images by analyzing tissue characteristics using magnetic fields and radio waves. It is the current imaging modality of choice due to its improved soft tissue differentiation, ability to detect diffuse axonal injury and lack of ionizing radiation compared to computed tomography (CT). The gross, macroscopic structural changes with CTE include cerebral atrophy that is most severe in the frontotemporal lobes, vermis, thalamus, mamillary bodies and hypothalamus. There is also ventricular enlargement, thinning of the corpus callosum and depigmentation of the substantia nigra and locus coeruleus. Though it is not a consistent feature of CTE, neuropathologic change (CTE-NC), i.e., the presence of cavum septum pellucidum in imaging, is associated with CTE. Microhemorrhages representing diffuse axonal injury may also be present [26]–[30]. These structural findings are not specific to CTE, however [30]. Therefore, there has been increasing research on the use of alternative, more advanced imaging methods as tools to identify and understand the progression of CTE in vivo.

## 2. Investigational imaging modalities

### 2.1. Quantitative Structural Analyses

Methods of quantitative brain volume analyses, like assessing cortical thickness in other neurodegenerative diseases, have provided useful for diagnosis and prognosis [31]–[34]. A study showed that hippocampal volume in football athletes was inversely correlated with the presence of concussions and amount of football played [35]. These findings, however, may not be specific to CTE considering the volume loss seen in other neurodegenerative diseases [36]. Furthermore, the inclusion criteria consisted of sport participation within the last year in those aged between 18–26. Therefore, it did not necessarily include or correlate with the presence of neuropsychiatric symptoms associated with CTE [16].

### 2.2. Diffusion Tensor Imaging

Diffusion tensor imaging (DTI) is an MRI technique that examines the longitudinal diffusion of water through axons to evaluate the orientation and integrity of white matter tracts. A fractional anisotropy (FA) close to 1 means that diffusion occurs along one axis and is otherwise restricted. Axial diffusivity (AD) and radial diffusivity (RD) are similar measures that reflect the magnitude of diffusion running parallel and perpendicular to white matter tracts, respectively [37],[38]. As such, decreased FA, decreased AD and increased RA would be expected in CTE due to decreased white matter integrity. These findings have been demonstrated in mTBI, and even had prognostic value [39]–[47]. A post-mortem tissue DTI analysis of patients with confirmed CTE-NC by Holleran et al. demonstrated associations between decreased FA and reduced white matter integrity [38]. A DTI analysis by Herweh et al. that evaluated male amateur boxers demonstrated associations between decreased FA and neuropsychological outcomes [48]. A study by Kraus et al. showed that study subjects who were included on the basis of having a history of mTBI (22 subjects) or moderate to severe TBI (17 subjects) had decreased FA and RD. The study also suggested that DTI can help to determine the relationship between TBI and cognitive differences and distinguish the spectrum and severity of TBI [49]. Another study showed decreased FA and no changes in the RD and FA in patients who were football players with sub-concussive impacts, with return to baseline after they abstained from play. Further studies are needed to assess the utility of RD and AD in patients specifically with CTE [50].

### 2.3. Functional MRI

Functional MRI (fMRI) is also known as blood oxygen level-dependent MRI. Neuronal activation in specific brain areas results in an increased oxyhemoglobin-to-deoxyhemoglobin ratio secondary to increased local blood flow, resulting in changes in magnetic susceptibility that are detected by fMRI when a specific task is performed [51]. This method is heavily used in behavioral and physiologic research, as it correlates well with neuronal activity. A theoretical limitation is that the results may be confounded in patients with CTE who already have reduced and altered cerebral blood flow. This modality is yet to be investigated in CTE. A few studies have, however, demonstrated altered brain activation patterns in the fMRI results of living patients with acute and repetitive mTBI [52]–[62]. The correlation between fMRI and altered brain activation patterns in those with mTBI may overwhelm the theoretical limitation. Furthermore, arterial spin imaging MRI, a type of fMRI, has shown to represent aberrant cerebral blood flow in those with mTBI [63].

### 2.4. Magnetic Resonance Spectroscopy

Magnetic resonance spectroscopy measures concentrations of metabolites within the brain based on the chemical shift of their protons, which is determined by the proton's chemical environment. This modality is useful for CTE when considering the pathological changes, including neuroinflammation, in the acute and chronic stages of the disease. In fact, a study investigating male USA National Football League (NFL) players between 40–69 with self-reported neuropsychiatric symptoms were found to have decreased cellular energy metabolism, as evidenced by lower creatinine in the parietal white matter. Neuro-inflammatory metabolites like glutamate, glutathione and myo-inositol also correlated with their behavioral and mood symptoms [64]. Several other studies have demonstrated metabolite abnormalities in patients with a history of repeated head impacts, including decreases in NAA, NAA/Cho and NAA/Cr, as well as increases in Cho, ml, glutamine, choline, fucose and phenylalanine [65]–[70].

### 2.5. Susceptibility Weighted Imaging

Susceptibility weighted imaging (SWI) takes advantage of different responses, or susceptibilities, to molecules within a magnetic field. These susceptibilities are measured as phase shifts and superimposed on an MRI, highlighting local susceptibility changes. In the setting of TBI, it can be used to reveal hemorrhagic contusions or diffuse axonal injury. SWI abnormalities, including microhemorrhages, have been demonstrated in contact sport participants, active duty military members and those with concussive-like symptoms and a history of repetitive mTBI [71]–[74]. Considering that it has shown utility in predicting neuropsychiatric outcomes for those with acute mTBI, its use should be considered in predicting the likelihood of the development of CTE [75],[76]. Neurodegenerative disorders often have characteristic features and positions of cerebral microbleeds [77],[78]. Research investigating the distribution and features of cerebral microbleeds in CTE to make a specific diagnosis would be beneficial.

### 2.6. Position Emission Tomography

Position emission tomography (PET) CT, which employs the use of radioisotopic biomarkers, has been garnering interest for elucidating elevated tau, beta-amyloid, neurofibrillary tangles and other neuroinflammatory proteins [79]. For example, FDDNP binds to the neurofibrillary tangles and proteins that are associated with CTE. As such, it has been employed in the diagnosis of CTE [79],[80]. However, FDDNP has also been shown to bind to beta-amyloid and hyperphosphorylated tau and is therefore limited in specificity when discriminating against other neurological degenerative diseases, such as Alzheimer's disease [79].

The development of biomarkers specific for the hyperphosphorylated tau proteins associated with CTE, such as [^18^F]AV-1451 (flortaucipir), are of interest and have been recently studied. [^18^F]AV-1451 binds to the paired helical tau deposition associated with Alzheimer's disease, and studies are being conducted to investigate its utility for visualizing tau deposition patterns that are associated with CTE, such as those in the medial temporal lobe, brain stem and diencephalon [81]. One such study involved [^18^F]AV-1451-PET scans from 26 former NFL players aged between 40–69 with reported neuropsychiatric symptoms; the researchers observed a statistically significant increase in the mean standard reuptake of [^18^F]AV-1451 in the bilateral superior frontal, bilateral medial temporal and left parietal regions as compared to the controls [82]. Another study followed a retired NFL player who underwent an MRI and [^18^F]AV-1451 PET scan, which revealed uptake in the bilateral medial temporal lobes and parietal regions 4 years before a post-mortem diagnosis of stage-4 CTE [83]. These studies, which are confined to small sample sizes and the single case report, illustrate the need for further investigation to validate [^18^F]AV-1451 as an optimal radiotracer for in vivo PET scans to diagnose CTE. Other developing PET tracers of interest that bind to tau proteins include [11C]PBB3[84], THK-5105[85], THK-5117[86], THK-5351[87] and T807[88]. [^18^F]florbetapir and [^11^C]PiB PET measure amyloid beta plaques [89],[90]. The characteristics of selected biomarkers are reviewed in Table 1. Studies have also investigated the use of [^11^C]flumazenil and [^18^F]flumazenil, which bind to the GABA_A_ system in patients with a history of repeated head injury [91]. Lastly, the translocator protein (TSPO) and copper have also been targeted with several radiotracers to assess inflammation [91]. Several studies have also investigated the use of [^18^F]FDG for patients with a history of mTBI, generally showing decreased brain glucose metabolism in the cerebellum, vermis, pons, temporal lobe, prefrontal cortex and limbic system [91]. Though cortical uptake regions vary, studies have generally consistently demonstrated uptake in the temporal lobes, limbic system, midbrain, hippocampi and amygdalae [2],[79],[91]. The need for further research into PET biomarkers for hyperphosphorylated tau proteins specifically associated with CTE and tasked with reducing off-target binding is warranted.

**Table 1.** PET CT biomarkers.

Biomarker	Specificity
FDDNP	Affinity for intracellular neurofibrillary tangles but has been found to be “non-selective” due to its binding with extracellular β-amyloid and tau, which is a feature of Alzheimer's disease and not necessarily CTE [17],[79],[91]
[^18^F]AV-1451 (flortaucipir)	Affinity for hyperphosphorylated tau proteins. However, there is a possibility of false negatives, as there is also high binding affinity for paired helical tau filaments in Alzheimer's disease and not CTE [91],[92].
[^11^C]PBB3	Affinity for Alzheimer's disease tau pathology but has mixed reviews over its ability to identify paired helical tau filaments in CTE [93],[94]
THK-5105	High binding affinity to tau protein aggregates and tau-rich Alzheimer disease, but it has reported to have a high background signal in PET images, which could affect its utility. Also, it has not been investigated for tau proteins seen in CTE-related pathology [86],[95].
THK-5117	Affinity for Alzheimer's disease-related tau protein in the medial temporal lobe in port-mortem patients, but not yet investigated for the CTE-related tau deposits [96].
THK-5351	Showed affinity with increased t-tau levels in the parahippocampal gyrus, but not investigated for CTE-associated tau patterns to date [97].

## 3. Fluid biomarkers

### 3.1. p-Tau

CTE is characterized by an accumulation of differentiated cis p-tau proteins in the vasculature of sulcal depths with large groups of astrocytic tangles, neurofibrillary tangles and neurites [17]. These abnormal, non-functional p-tau clusters develop within axons [17],[98]. High values of p-tau are also found in single-event TBI and other neurodegenerative diseases, and therefore cannot serve as the sole means of diagnosis of CTE [99],[100]. However, it can serve as a biomarker when considered in the overall clinical setting with accompanying imaging findings from developing diagnostic tools, like PET.

### 3.2. TREM2

A promising biomarker that is not as widely employed for tau is the inflammatory marker triggering receptor expressed on myeloid cells 2 (TREM2), a triggering receptor found in several myeloid lineage cells such as peripheral macrophages, dendritic cells and microglial cells in the central nervous system (CNS) [101]. TREM2 is also expressed in the microglia of the brain, regulating microglial activation and playing a multi-faceted role in its immune response [102],[103]. Animal studies have shown that TREM2 is upregulated in the early stages following injury, making it a potential biomarker for TBI and other head injuries [104]. When microglia in the brain are activated, following injury, cleavage of TREM2 by proteases follows. These proteases release soluble TREM2 (sTREM2), indicating that injury has occurred. A study highlighted the relationship between sTREM2 levels in cerebrospinal fluid (CSF) and t-tau concentrations in 68 former NFL players aged 40–69 with self-reported neuropsychiatric symptoms (compared to healthy controls), ultimately finding a positive correlation between the two [102]. The presence of TREM2 variants increases the likelihood of developing Alzheimer's disease by 2–4 times [105],[106]. Therefore, elevated levels of sTREM2 are non-specific. Because upregulation of TREM2 begins early and persists over time [104], it could prove to be a key inflammatory marker used for the diagnosis of CTE in the appropriate clinical context.

### 3.3. CCL11

A biomarker with potential for pre-mortem CTE diagnosis is the chemokine C-C motif chemokine ligand 11 (CCL11). Chemokines are proteins that play a central role and facilitate biochemical and cellular events in the immune response. They upregulate leukocytes and act as secondary pro-inflammatory mediators [107],[108]. CCL11 is a chemoattractant of eosinophils in the peripheral immune system and has recently been shown to also penetrate the blood brain barrier [107]. A study showed that the brain of mice secreted CCL11 as a response to the inflammation of astrocytes, pericytes and microglia [109]. A key study showed an increase in the plasma blood levels of CCL11 to correlate with a decrease in learning, memory and neurogenesis in the brains of mice [110]. It has been proposed that the main reason for CCL11's involvement in neurological decline is its ability to increase the microglial production of reactive oxygen species and promote excitotoxic neuronal death [111]. A study indicated that CCL11 is released in the CSF from the choroid plexus in the brain, suggesting direct effects on the brain [112]. This is also associated with an increase in the ratio of cytokine interleukin (IL)-4 and interferon (IFN)-γ in the choroid plexus and CSF. Prior research has shown the physiological importance of CCL11 to neurological function, but it may be a useful biomarker for CTE considering its ability to distinguish it among other neurodegenerative diseases. A collection of studies showed that plasma CCL11 increased in patients with Alzheimer's disease and Huntington's disease, while it decreased in amyotrophic lateral sclerosis and secondary progressive multiple sclerosis [113]–[115]. Using ELISA, a post-mortem study of 23 former football players with neuropathologically diagnosed CTE and 50 subjects with neuropathologically diagnosed Alzheimer's disease showed a statistically significant increase in the CCL11 levels in the dorsolateral frontal cortex of CTE subjects compared to the Alzheimer's disease and control subjects [116]. Another study with subjects aged 25–33 showed significant increases in CCL11 in the CSF of retired football players relative to swimmers with no TBI history and a sedentary control group. Analysis of the IL-4-to-IFN-γ ratio also showed significant increase in the football players compared to the others in the study. Lastly, CCL11 levels showed a strong positive correlation with the number of years of football played [107]. There is a lot of promise that CCL11 can provide CTE diagnosis for patients pre-mortem. More comprehensive research can be done to analyze the relationship of CCL11 levels with the IL-4-to-IFN-γ ratio found in the CSF of different types of neurodegenerative diseases in pre- and post-mortem brains to build a more accurate predictive model for diagnosis.

### 3.4. NFL

Another biomarker widely employed in TBI is neurofilament-L (NfL). NfL comes from the intermediate filament protein family and is part of the neuronal cytoskeleton [117]. It can serve as an indicator of CNS axonal damage. NfL is released into the CSF [117]. A meta-analysis with a sample size of 1118 patients showed that NfL CSF, serum and plasma levels were significantly higher in patients with TBI compared to the control patients without prior TBI [117]. Another study showed that patients with Alzheimer's disease, Guillain-Barré-syndrome and amyotrophic lateral sclerosis had increased levels of serum NfL compared to a control without CNS damage [118]. These studies suggest that NfL could be used to also conduct future research toward CTE diagnosis.

### 3.5. Glial fibrillary acidic protein

Glial fibrillary acidic protein (GFAP) is a cytoskeletal monomeric filament protein located in the astroglial cells of gray and white matter [119]. A study showed that levels of GFAP, tau and NfL were all higher in the group of 277 patients suspected with mTBI, with GFAP yielding high discriminatory power in differentiating these patients from the 49 healthy controls, with an area under the curve (AUC) of 0.93 [120]. In the same study, GFAP similarly served as a strong predictor of mTBI when examining MRI abnormalities, with an AUC of 0.83 [120]. Another study of GFAP's ability to predict CT abnormalities showed an AUC of 0.88 when examining 215 patients (83% with mTBI; mean age 42.5 ± 18.0) [121]. Further analysis could be done using pre-mortem CTE subjects and comparing GFAP levels in those patients to a control to assess whether this translates to specifically diagnosing CTE over other diseases as a result of a TBI. The characteristics of the biomarkers are summarized in Table 2.

**Table 2.** Blood biomarkers for the diagnosis of CTE.

Biomarker	Description	Significance	Advantages	Limitations
p-tau	Hyperphosphorylated tau protein found in the cortical vasculature within the sulcal depths [17].	-Repetitive head injury causes the conversion of typical tau protein to p-tau [2]. -P-tau is indicative of axonal functional decline and will deposit in predictable patterns and high concentrations following brain injury [17],[122],[123].	-Consistent and sensitive results considering large concentrations following injury [123]. -A blood sample is less invasive than lumbar puncture [124].	Other neurodegenerative diseases also express high concentrations of p-tau [123].
TREM2	Triggering receptor found in myeloid lineage cells that regulates CNS microglial activation [101].	-Cleavage of TREM2 by proteases following head injury produces sTREM2 -Increased sTREM2 levels in the CSF are indicative of increased protease activity, likely due to trauma [102].	Early upregulation of TREM2 following head injury may eventually lead to its diagnostic use in potential CTE cases in vivo [102],[104].	-Invasive sample collection with lumbar puncture [102]. -TREM2 variants can impair the function of receptors due to poor signaling, ultimately leading to decreased sTREM levels [125].
CCL11	-Chemokine that serves as a mediator in inflammatory cascades [107],[108]. -Penetrates the blood brain barrier [107]. -Secreted into CSF by choroid plexus in the brain [112].	-Increases microglial production of reactive oxygen species and promotes excitotoxic neuronal death [111]. -Reflective of neuroinflammatory processes [111].	-Potential ability to differentiate between CTE and other neurodegenerative diseases [116]. -Possible correlation with number of repeated head impacts [107].	-Its main role in the CNS is unclear, as it is a chemoattractant of eosinophils in the peripheral immune system [112].
NfL	Part of the Intermediate filament protein family and of the neuronal cytoskeleton [117].	-Can be measured, as axonal damage induces its release into the CSF [117].	-Released in a delayed fashion and may be correlated with cognitive decline in patients with chronic TBI [126]. -Specific to CTE [116].	-Conflicting data regarding its validity in accurately diagnosing CTE [117].
GFAP	Cytoskeletal monomeric filament protein in the brain's astroglial cells [119].	-Reflective of astroglial injury and released acutely following TBI [116].	-Better predictor of mTBI than NfL and p-tau [120].	-Utility in CTE specifically unknown [120],[121].

## 4. Conclusion

In conclusion, the current clinical diagnosis of CTE relies on clinical symptomatology and structural imaging findings. While there is extensive research on imaging and fluid biomarkers in relation to TBI, there is comparatively limited research on CTE. Particularly, the referenced studies are often small and investigate mTBI, TBI or repetitive head injury and do not study CTE directly. Investigating CTE directly is especially difficult considering the lack of consensus on pre-mortem diagnostic criteria. The studies also frequently include patients based on a history of sport participation alone, self-reported history of TBI or self-reported neuropsychiatric symptoms. The studied biomarkers are also often elevated in other neurodegenerative disorders, and there is relatively limited research on the use of biomarkers to differentiate CTE from other neurodegenerative disorders. Lastly, many of the fluid biomarkers are also elevated following a single TBI event, and there is not an imaging or fluid biomarker approved solely for CTE. We look forward to further research on the early promising imaging modalities and fluid biomarkers to potentially assist in the diagnosis of CTE and in differentiation of it from other neurodegenerative diseases.
